# Draft Genome Sequence of the Novel Thermoacidophilic Archaeon *Acidianus copahuensis* Strain ALE1, Isolated from the Copahue Volcanic Area in Neuquén, Argentina

**DOI:** 10.1128/genomeA.00259-14

**Published:** 2014-05-08

**Authors:** M. Sofía Urbieta, Nicolás Rascovan, Camila Castro, Santiago Revale, M. Alejandra Giaveno, Martín Vazquez, Edgardo R. Donati

**Affiliations:** a CINDEFI (CCT La Plata-CONICET, UNLP), Facultad de Ciencias Exactas, Universidad Nacional de la Plata, La Plata, Argentina; b Instituto de Agrobiotecnología de Rosario (INDEAR), CONICET, Predio CCT, Rosario, Argentina; c IDEPA (CCT Comahue-CONICET, UNCo), Departamento de Química, Facultad de Ingeniería, Universidad Nacional del Comahue, Neuquén, Argentina

## Abstract

*Acidianus copahuensis* is a recently characterized thermoacidophilic archaeon isolated from the Copahue volcanic area in Argentina. Here, we present its draft genome sequence, in which we found genes involved in key metabolic pathways for developing under Copahue’s extreme environmental conditions, such as sulfur and iron oxidation, carbon fixation, and metal tolerance.

## GENOME ANNOUNCEMENT

Copahue is a naturally acidic geothermal area located in the northwest of Neuquén province in Argentina and dominated by the Copahue volcano. The constant volcanic activity has determined the acidic, sulfur-rich, and high-temperature environments found in Copahue, such as Río Agrio and the geothermal springs (1, 2). Biodiversity studies done in Copahue (3, 4) revealed the presence of acidophilic bacteria and archaea, some of which are capable of growing at a high temperature and of using iron or sulfur compounds as energy sources. The detection of these extremophiles, most of which are yet uncharacterized and apparently autochthonous, makes Copahue a rich source of novel species with potential applications in biotechnological processes, mainly those related to biomining.

One such species is *Acidianus copahuensis*, an archaeon from the *Crenarchaeota* phylum, isolated from various hot springs in Copahue (5). *A. copahuensis* is a facultative chemolithoautotrophic and thermoacidophilic microorganism (optimal growth conditions, 75°C and pH 2.5). It is capable of autotrophic growth, both aerobically by using iron and different sulfur compounds as energy sources and anaerobically by using H_2_ and S as electron donors and Fe(III) or S as final electron acceptors. It can also grow heterotrophically by using yeast extract or glucose (5).

The genome sequence of *A. copahuensis* strain ALE1 was obtained using a whole-genome shotgun (WGS) strategy with a 454-FLX Titanium pyrosequencer at INDEAR, Argentina. Assembly was done using Celera Assembler version 7.0, with 52× genome coverage, which generated a total of 68 contigs. The draft genome is 2,454,023 bases in length. The G+C content of the genomic DNA is 35.63 mol%. Genome annotation was done using the RAST server based on subsystem descriptions (6). A total of 2,548 coding sequences (CDSs) and 52 structural RNAs (49 tRNAs and 3 rRNAs) were predicted. Forty-seven percent of the CDSs were classified as hypothetical proteins and 20% as known enzymes. Thirty-four percent of the CDSs were assigned to RAST subsystems.

The genome of the *A. copahuensis* ALE1 strain presents genes that might be related to the relevant metabolic features of the strain. Key enzymes for sulfur compounds oxidation, such as sulfur oxygenase-reductase (SOR) and thiosulfate:quinone oxidoreductase (TQO), were detected. Homologs of some of the Fox cluster enzymes, associated with iron oxidation, were also found. The genome presents proteins of the five major terminal oxidase complexes of *Sulfolobales*, so far reported in only two species (7). Carbon fixation through the 3-hydroxypropionate–4-hydroxybutyrate cycle can be inferred by the presence of key enzymes of this pathway. An interesting discovery is the presence of *aioAB* genes encoding arsenite oxidase, which might be an indication of a bioenergetic use of arsenite. These genes were reported in *Acidianus hospitalis* and *Sulfolobus tokodaii* genomes but not in other *Sulfolobales*. In an all-versus-all BLASTp comparison (e value, <1e^-20^) to *A. hospitalis* (the closest sequenced relative), *A. copahuensis* showed 789 unique proteins, while in a comparison with *Metallosphaera sedula* (another related archaeon with very similar metabolic features), 766 unique proteins were detected.

### Nucleotide sequence accession number.

This WGS project has been deposited at DDBJ/EMBL/GenBank under the accession no. JFZT00000000.
